# Wear Your Heart on Your Sleeve: Smart Textile ECG Wearables for Comfort, Integration, Signal Quality and Continuous Monitoring in Paroxysmal Atrial Fibrillation

**DOI:** 10.3390/s25030676

**Published:** 2025-01-23

**Authors:** Alexandra E. Avanu, Gianina Dodi

**Affiliations:** 1 Faculty of Medicine, Grigore T. Popa University of Medicine and Pharmacy of Iasi, 700115 Iasi, Romania; 2 Faculty of Medical Bioengineering, Grigore T. Popa University of Medicine and Pharmacy of Iasi, 700454 Iasi, Romania; gianina.dodi@umfiasi.ro

**Keywords:** atrial fibrillation, stroke risk, wearable, smart textile, ECG monitoring, electrochemical sensors, continuous monitoring

## Abstract

Atrial fibrillation (AF), a prevalent cardiac arrhythmia and a major contributor to stroke risk, is anticipated to increase in incidence with the aging global population. For effective AF management, particularly for paroxysmal AF (PAF), long-term and accurate monitoring is essential. However, traditional monitoring methods, including Holter ECGs and implantable cardiac monitors (ICMs), present limitations in comfort, compliance and extended monitoring capabilities. Recent advancements in wearable technology have introduced smart textile-based ECG devices, which incorporate electrochemical sensors into fabrics, enabling non-invasive, continuous monitoring while enhancing user comfort. This review evaluates textile-based ECG devices by comparing their performance—assessed through AF detection rates, signal-to-noise ratio (SNR) and total analysis time—against conventional Holter monitoring and the 12-lead ECG. Furthermore, this review examines user acceptability factors, including patient-reported comfort, usability during resting and physical activities and skin-related adverse effects. The findings aim to provide insights for future device development and facilitate their integration into clinical practice.

## 1. Introduction

According to the World Health Organization (WHO), cardiovascular diseases (CVDs) are the leading cause of death and disability in Europe, claiming 10,000 lives each day and representing over 42.5% of annual fatalities [1]. Atrial fibrillation (AF), the most common cardiac arrhythmia and a significant risk factor for stroke, contributes significantly to morbidity and mortality among older adults [2]. With the aging population and increased comorbidities, AF prevalence is expected to double in the coming decades [3]. Despite significant advancements, the diagnosis is often underestimated in clinical settings [4] and patient management remains inadequate [5]. Electrocardiography (ECG) is the primary diagnostic tool for detecting all forms of AF. However, long-term monitoring with traditional ECG methods is not feasible outside the hospital setting. This limitation is particularly critical for paroxysmal AF (PAF), which often spontaneously resolves within seven days, but may require continuous monitoring to detect intermittent episodes and guide appropriate management [6]. Due to its randomly occurring nature, PAF poses great diagnostic challenges [7]. Many cases are found only after an episode of cryptogenic stroke, which consequently delays the initiation of therapy [8]. The STROKE-AF randomized clinical trial (RCT) (NCT02700945) provided compelling evidence for this argument. AF was identified in 12.1% of stroke patients using continuous monitoring, compared to only 1.8% with standard care [4]. In another RCT (NCT04563572), enrolling 98 patients with PAF, it was anticipated that 75% of participants would experience detectable episodes during a 48 h ECG monitoring period [9]. However, only 27% demonstrated such occurrences, indicating that this duration might be insufficient to effectively capture PAF episodes.

Currently, long-term ambulatory rhythm monitoring options include external cardiac event recorders, which lack continuous monitoring and often suffer from patient compliance issues [10] and implantable cardiac monitors (ICMs), which can monitor up to three years, but while effective and miniaturized, they are invasive, carry some risks and have limited recording capacity [11]. The importance of extended monitoring was further highlighted in the pivotal CRYSTAL-AF (NCT00924638) [12] and EMBRACE-AF (NCT00846924) [13] studies, that found subclinical AF in 12–16% of participants over 3–12 months using an ICM and an external non-looping event recorder, respectively. These results advocate for long-term monitoring in all patients with cryptogenic stroke. Other non-invasive options include 24–48 h Holter monitoring, which offers high signal quality and specificity, yet has limited sensitivity and generally low diagnostic yield [10], with low signal-to-noise ratio (SNR) caused by motion, muscle artifacts and overlapping stronger ventricular signals [14,15]. Their bulky designs, with conventional Ag–AgCl electrodes, reliance on wires and skin-irritating and allergenic electrode gels, contribute to discomfort and fluctuating contact resistance [14,16]. Other factors such as respiratory movements or changes in electrode impedance due to perspiration are also important contributors to baseline instability in ECG recordings [16].

As a result, new wearable ECG sensors have been developed in recent years, using methods like impedance wave measurements in smartwatches or mobile apps [17]. However, most current systems only track heart rate (HR) without ECG tracings, with a lack of validated data available on their signal quality. One prominent option is the 14-day, three-lead Holter ECG by Norav, featuring a compact design that promises superior signal quality and user-friendly features [18]. Since the median time to detect PAF is 84 days [19], researchers have explored as well, integrating ECG sensors into textiles, leading to the development of smart textile-based ECG devices [20,21,22]. These fabrics are designed to integrate the comfort of conventional textiles, which lowers manufacturing costs, with the functionality of embedded electronic components, allowing for ECG and HR monitoring, while ensuring durability, reliability, ergonomics and ease of use [20,23]. Embedding the leads in clothing should offer a non-invasive solution for continuous monitoring, without user input [24]. These devices aim to overcome the location limits of traditional medical equipment, allowing doctors to manage patients’ health remotely via telemedicine [16,20,25,26].

However, practical implementation faces challenges, particularly in integrating electronic components, as current connections between electronics and textiles lack the durability required for commercial viability [27]. This review explores the latest technologies used in textile-based ECG wearable devices to diagnose PAF, highlighting the integration challenges, performance of electrochemical sensors and future directions to improve sensor accuracy and patient comfort.

## 2. Materials and Methods

A comprehensive search of the literature was performed using PubMed, Embase (Ovid), Medline (Ovid) and CT.gov, from 2017 to 2024. The search strategy included keywords related to ECG monitoring using textile electrochemical sensors, e.g., textile sensors, fabric-based sensor, sensor-equipped textile, textile electrode, fabric electrode, fabric-integrated electronic, e-textile sensors, smart garment, smart textile, smart clothing, textile-based electronic, fabric-integrated electronic, electrochemical sensors, wearable electronic device, ECG monitoring, ECG tracing, portable ECG, continuous ECG, Holter monitor, PAF, intermittent AF, as well as other relevant synonyms and Medical Subject Headings (MeSH) terms.

The inclusion criteria were as follows: (1) peer-reviewed articles involving (2) patients aged 60 years or older, (3) with or without a diagnosis of AF, defined as a sustained episode lasting 30 s or more, in accordance with the European Society of Cardiology’s diagnostic criteria for clinical AF [7], independently validated by expert cardiologists to ensure accuracy, reliability and clinical credibility assessing (4) the integration of textile ECG sensors in terms of usability, accuracy and/or durability (mandatory washability of the textile and incorporation of dry electrode technology, to ensure compliance with modern healthcare standards), with no language restrictions. The performance metrics employed in this review include AF detection rates, signal-to-noise ratio (SNR) and total analysis time. Although additional parameters, such as signal stability, sensitivity and specificity for AF detection were also considered, limited information was available on these factors. Furthermore, user acceptability was explored, with particular focus to patient-reported comfort, device usability during both resting and physical activities and the occurrence of skin-related adverse effects, including redness, rash and skin chafing. We excluded studies that involved adults younger than 60 years and those using non-ECG wearables or photoplethysmography, as well as studies lacking clinical testing or utilizing wet electrodes with non-washable textiles.

**Figure 1:**
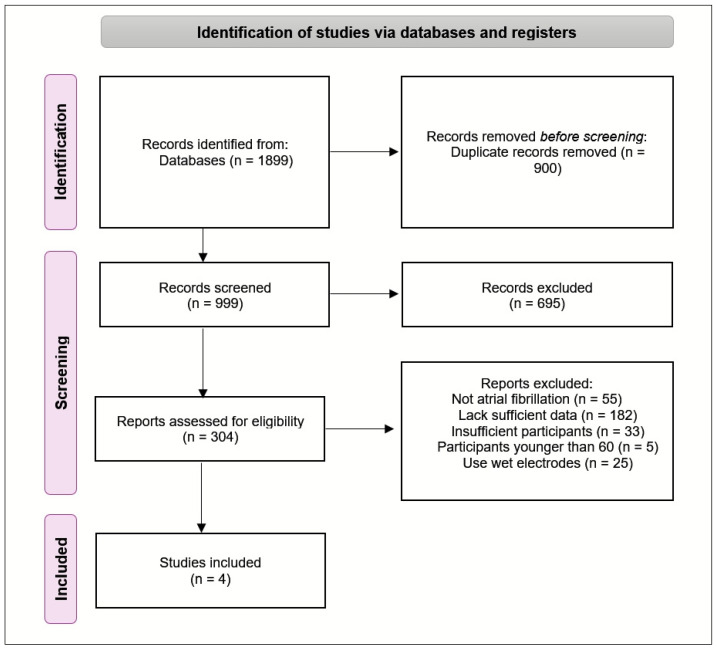
PRISMA flow diagram.

Two independent reviewers screened the titles and abstracts to determine initial eligibility, followed by a full-text assessment of selected studies to ensure they met the predefined inclusion and exclusion criteria. Data extraction was performed using a standardized form, capturing details on study design, sample size, characteristics of the devices, outcomes related to signal quality, user comfort, technical performance and limitations. The findings are presented narratively, supplemented by tables and figures summarizing key features of the included studies. The authors checked the reference lists of all included studies for any relevant additions. We identified 1899 studies in total, of which 4 studies, encompassing 3262 participants, were included. These studies were primarily observational and were published between 2022 and 2023. To provide an overview of the study selection process, a PRISMA flowchart [28] is included in Figure 1. To ensure a thorough understanding of the existing literature on wearable textile ECG sensors, within the same timeframe, we identified 15 reviews and systematic reviews throughout our search. Of these, four were directly relevant to the focus of this study and will be discussed in detail.

Due to the broad scope of the topic and its varied applications in cardiovascular research, a literature review was identified as the most appropriate approach, serving as a foundational step and a precursor toward a future systematic review. To evaluate the quality and credibility of the included studies and their various study designs, we used the Medical Education Research Study Quality Instrument (MERSQI) score, as shown in Table 1. 

## 3. Past and Current Developments

This review aims to build upon the existing body of literature on textile-based wearable ECG monitoring systems, with a specific focus on AF management through wearable technology. Below, we summarize key findings from previous analyses that contributed to the understanding of wearable ECG devices.

The 2019 review by Soroudi et al. [31] focused on the impact of electrode placement in ECG smart garments, specifically on the influence on the quality of biosignal sensing. Several trials were analyzed, including those using the WearItMed smart shirt for individuals with neurological disorders, as well as a smart shirt with printed electrodes positioned at the Wilson Monopolar Precordial lead monitoring sites. Through this analysis, they identified key factors, essential for achieving high-quality ECG signals while ensuring comfort and wearability of the garment. A year later, Singhal and Cowie et al. [32] discussed the role of wearables in heart failure, including a section dedicated to innovative AF monitoring systems. They highlighted two FDA (Food and Drug Administration) approved wearable patches, Carnation™ and VivaLNK™, as well as the smart textile vest Cardioskin™. Additionally, they examined the Apple™ Heartline Study (NCT04276441), a randomized, decentralized trial involving US participants aged 65 and older.

In 2021, a systematic review [29] further explored the use of e-textiles for clinical applications, with 21 papers related to ECG since 2010. The studies examined various e-textiles, including a novel cloth with conductive multiwalled carbon tubes (MWCNTs) and silver/silver chloride electrodes; an elastic armband based on nylon graphene oxide-coated fibers; an elastic bandage composed of Ag particles, MWCNTs, silicone rubber and stretchable conductive adhesive (SCA) electrodes deposited on a polydimethylsiloxane (PDMS) layer; a silk sericine–MWCNT composite ink for conductive fibers/yarns and textiles; commercial patches of silver nanowire/thermoplastic polyurethane (AgNW/TPU) electrodes; sportswear with EMG sensors, a strain sensors and a fluoroelastomer conductor, reinforced with polyvinylidene fluoride (PVDF) nanofibers; two prototypes of sensing vests with different combinations of conductive and non-conductive yarns; a novel shirt prototype made of three electrodes knitted with Elitex; a custom-made laminar smart textile electrode of a silver woven fabric potentially integrated in a shirt and a sock; and so on. An important diagnostic example, with quality levels comparable to conventional Holter, was a T-shirt working as a 12-lead ECG acquisition system (electrodes made of silver yarns and hydrogel pads). Nevertheless, the review noted that these products were primarily evaluated on healthy subjects in small sample sizes.

In 2023, Dahiya et al. [33] conducted a scoping review that described the landscape of continuous ECG monitoring systems focusing on device accuracy, signal quality, comparability and visual assessment. Out of the 12 identified relevant studies, 6 were focused on healthy adult subjects, with three examining controlled groups with AF. Notably, the Zio patch was used on 45 elderly patients, the Kardia Band with an Apple Watch provided single-channel ECG and the wearable chest-strap linked with the RITMIA app successfully detected AF in 95 patients, demonstrating 97% sensitivity and 95.6% specificity.

In this context, to the authors’ knowledge, this review is the first to chart the usability and technical specifications of smart textile wearables for ECG monitoring across an PAF population.

## 4. Performance and Integration

There are limited methods to integrate new technology into devices that are simple, affordable and low-effort. Recent findings from implantable cardiac devices and wearable sensors suggest that AF should not be viewed as simply present or absent when assessing stroke risk [10]. Instead, the risk appears to increase with AF burden, which refers to the amount of time a person spends in AF. Continuous monitoring devices can measure AF burden by tracking the frequency and duration of AF episodes or the percentage of time spent in AF. Studies have shown that episodes lasting over 24 h or AF burden exceeding 5.5 h per day are linked to a higher stroke risk [34]. Higher AF burden is also associated with increased risk of heart failure and mortality.

### 4.1. Technical and Performance Indicators

The wearable technology industry, particularly in the realm of “smart textiles”, has emerged as a key sector combining flexible electronics, technical textiles and advanced material science. These systems typically include garments with embedded sensors, data processing hardware, wireless transmission and a remote monitoring station to continuously track vital signs [16]. Wearable textile devices have evolved from bulky and rigid to thin and highly miniaturized designs [27]. Sensors are directly integrated into shirts, belts, vests and other items in order to raise patient compliance [16]. These devices are less obtrusive, improving the user–machine interface [27]. As technology advances, the goal is to seamlessly integrate such devices into daily life. To that end, the user–machine interface is becoming more intuitive, with wireless systems playing a critical role in monitoring vital signs and motion for operational convenience.

For effective long-term monitoring, the electrodes used in textile-based ECG wearables are critical in determining the quality of the acquired signal [35]. To meet this need, they must be flexible, which allows for better body conformity than rigid disk electrodes, biocompatible, lightweight, comfortable and capable of maintaining high SNR over extended periods [23]. Dry electrodes, which do not rely on conductive gel, offer the advantage of not drying out over time. These electrodes can be made from fabric or flexible materials like elastomers [36]. However, these often face challenges such as high contact impedance and polarization voltage when attached to the skin, leading to discomfort and susceptibility to noise interference [37] due to the absence of a gel layer [38]. These electrodes’ electrochemical characteristics, particularly skin–electrode contact pressure (SECP), are critical for achieving optimal performance [37]. The presence of sweat accumulated at the electrode–skin interface can help reduce it, but it is not uniform and does not provide the same ionic concentration as the gel, which can lead to inconsistencies in the signal quality by being more susceptible to motion artifacts.

**Figure 2:**
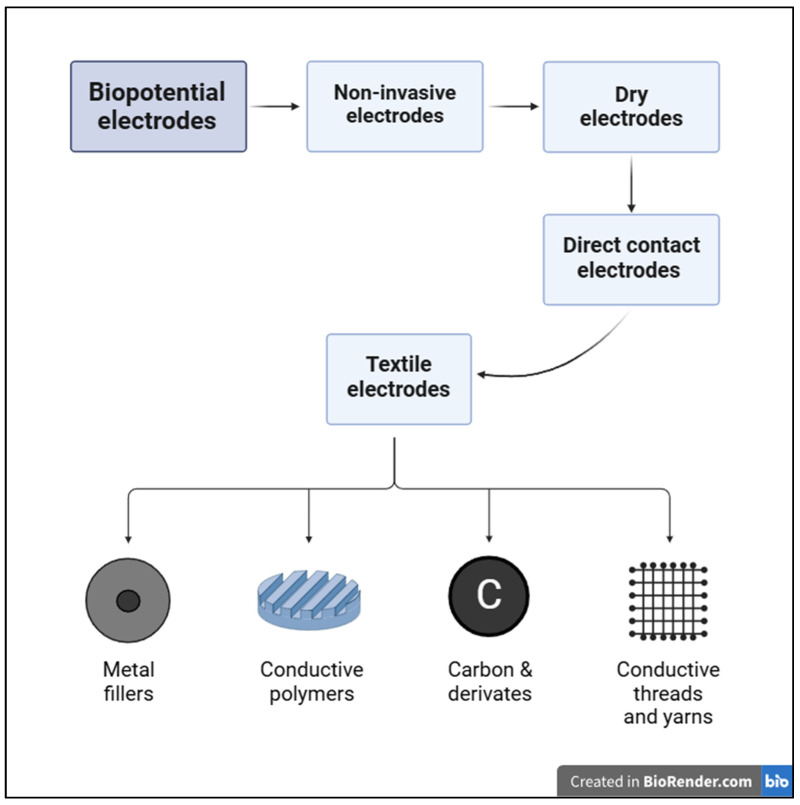
Classification of smart textile-based electrodes.

Various types of dry contact electrodes include metal electrodes, polymer electrodes and smart textile electrodes [38,39]. While metal electrodes offer better conductivity, they are rigid and do not conform well to the skin, leading to potential motion artifacts and lower SNR. Polymer electrodes, such as those made from PDMS or polyurethane, offer better conformance with the skin and are flexible, making them a good choice for wearable ECGs. Smart textile electrodes are characterized in Figure 2.

To ensure accurate diagnosis and appropriate management, the ESC recommends using standard 12-lead ECGs or single- and multiple-lead devices that provide ECG readings and advises against non-ECG wearables and those using photoplethysmography [7,40].

Table 2 and Table 3 present demographic and technical data from the four studies included in this paper. Most studies focused on geriatric patients, with a higher proportion of male participants, aged 65 and older. The exception is Machino et al. [30], which included patients with a mean age of 63.1 ± 10.6. The studies also report varying proportions of AF subtypes, with Pagola et al. [29] showing a higher incidence of paroxysmal AF cases.

Machino et al. initially conducted a prospective observational pilot study enrolling 18 patients, comparing a smart bra ECG wearable with a Holter ECG over three hours of continuous monitoring [11]. The electrode featured woven nanofibers coated with a conductive polymer, poly(3,4-ethylenedioxythiophene) poly(styrenesulfonate) (PEDOT-PSS), connected by an insulated electroconductive lead ribbon for signal transmission. PAF was detected in eight patients (44%); however, noise signals were more frequent in the wearable ECG, fragmenting AF episodes. The noise increased by 0.6% in the wearable due to reduced R-wave amplitude, leading to a 3-min (0.6%) AF episode interruption. This discrepancy was deemed negligible for long-term AF monitoring, as the flexible hitoe^®^ electrode and bra design with Velcro ensured consistent skin–electrode contact.

Later on, Machino et al. enrolled 67 patients, who had undergone initial AF ablation in an open-label randomized crossover study (RCT 032180018), 35 of whom used the textile wearable, for 14 days of monitoring [30]. Registered as a medical device in Japan, the electrode, sewn into sportswear fabric, provided continuous, non-invasive monitoring and was easily washable. They used the same polymer (PEDOT-PSS) and nanofiber electrodes, featuring pre-installed lead wires and pressure adjustment for comfortable, extended monitoring. Compared to the Holter monitoring system, the Garment ECG detected AF recurrence in 18% of patients, a significant increase from the 6% detected using the conventional method. While the classic Holter achieved a higher acquisition rate (lower noise level) of 100% compared to 82.4% for the Garment ECG, the latter offered a substantially longer total monitoring duration of 11 days vs. 1 day median. Despite its lower acquisition consistency, it contributed to the increased AF recurrence detection. The longer monitoring period of the Garment ECG enabled the detection of AF episodes at non-uniform intervals, including asymptomatic cases, which were often missed.

In 2023, a prospective multicenter study, by Pagola et al., monitored 224 patients with cryptogenic stroke over a 90-day period using a textile-based wearable device using elastomeric polymers that can stretch in two different directions or axes [29]. Of these, 72.76% (163 patients) completed the study, with early dropouts primarily attributed to device-related technical issues and patient non-compliance, particularly during the first month of monitoring. The technical difficulties, likely due to the device being used for extended periods, raise concerns about its feasibility for long-term use. Additionally, the device required approximately one hour of charging per day. The median monitoring duration exceeded 500 h across all study periods (30/60/90 days). Detection rates for PAF were 23.36% at 30 days, 6.29% from day 31 to day 60 and 5.71% from day 61 to day 90. The cumulative incidence of PAF detection increased to 35.87% by 90 days. Notably, the majority of PAF episodes were detected within the first month.

Finally, the 2023 NOMED-AF TECH cross-sectional study (ClinicalTrials.gov; NCT03243474) evaluated the use of a smart vest with two integrated ECG leads, two interchangeable recorders and a docking station, for ECG monitoring in order to identify high-risk patients with AF [17]. While one recorder was in use, the other was recharged, ensuring continuous monitoring. The average ECG monitoring period was 21.9 days. PAF was detected in 46 participants (69%), on average after 4 days of monitoring and the incidence of PAF rose from 1.9 after 24 h of monitoring to 6.2% after four weeks, demonstrating that extended monitoring led to a substantial increase in the detection of previously undiagnosed PAF. Despite some technical challenges—1.33% of participants experienced signal acquisition problems and 2.96% had less than one day of effective ECG recording—72% continued monitoring beyond 14 days. Artifacts were present in 4.2% of recordings, yet the signal quality remained high, with 96.2% of ECG data suitable for analysis. The study also acknowledged the potential for underestimating AF prevalence, as patients monitored for shorter periods, without detected AF, might have been diagnosed if monitored over longer periods of time, a finding demonstrated by the study’s results. PAF was detected in 46 (69%) participants, and the incidence of PAF rose from 1.9 after 24 h of monitoring to 6.2% after four weeks, demonstrating that extended monitoring led to a substantial increase in the detection of previously undiagnosed PAF. All devices’ performance indicators are summarized in Table 4.

Additionally, other wearable ECG devices deserved our attention, such as the LIVMOR AF Detection System, which received FDA approval in October 2021 as the world’s first medically authorized wearable for continuous cardiac monitoring [42]. This system utilizes a Samsung smartwatch linked to a cloud-based platform, enabling passive, continuous monitoring and making it ideal for asymptomatic patients or those at risk of AF. Many other wearable devices have not yet been validated for elderly stroke patients. Additional wearables, including the eMotion Faros and Suunto Movesense, are also being tested for accuracy.

### 4.2. Usability and Compliance

User compliance pertains to the degree to which individuals can effectively and comfortably interact with a device as designed, which is particularly crucial in medical applications. Ensuring consistent and sustained usage is essential for accurate data acquisition and the achievement of meaningful clinical outcomes.

The wearable utilized by Machino et al. showed minimal usability issues, with only a single case of skin contact failure reported and no other significant skin-related complications [11]. However, the study lacked comprehensive patient feedback and a larger sample size, which would have strengthened the findings. In contrast, their subsequent device, the Garment ECG, demonstrated a strong preference among patients, with 79% favoring it over the traditional Holter device [30]. Despite minor challenges with garment removal and a 2.3% incidence of skin redness, the device’s extended wearability and the absence of severe adverse events underscore its practicality and user-centered design for long-term monitoring. Additionally, with no reported cases of erosion or scarring, patients found the device significantly more comfortable compared to the traditional 24-h Holter monitor.

Pagola et al.’s device faced notable usability challenges, including technical issues and patient non-compliance, resulting in a 27% dropout rate over a 90-day protocol [29]. Structured follow-ups and professional application during hospital stays helped mitigate these challenges to some degree. However, questions remain regarding the device’s scalability for broader outpatient use, its reliability in less controlled environments and the long-term impact of these challenges on overall patient adherence and clinical outcomes. 

Ultimately, the NOMED-AF TECH clinical trial device was designed with an emphasis on user feedback, resulting in 82% of patients reporting no adverse effects during mid-term evaluations, while adverse events in the remaining cases were mild to moderate [17]. Participants, including those with disabilities, were encouraged to wear the vest frequently, even during physical activity, which contributed to its comprehensive usability assessment. Approximately 16.4% of users experienced mild skin irritation, such as redness, rash or chafing. Despite these minor issues, the device demonstrated satisfactory usability, with an average discomfort rating of 3.6–3.8 out of 10 and showcased a patient-friendly design well suited for long-term monitoring.

Table 5 provides a detailed stratification of usability parameters across the devices, offering a comparative perspective on comfort, adverse effects and overall user experience.

The presence of missing data in several key metrics across the studies points to a lack of standardization in how wearable ECG devices were tested and also different outcomes. Variations in the reporting of technical parameters, such as signal quality or sensitivity, complicate efforts to draw comprehensive conclusions about device effectiveness. This gap highlights the potential for improvement in future research, as standardizing the measurement and reporting of these parameters would provide clearer insights into the accuracy, reliability and patient comfort of different devices.

Moreover, the absence of essential data limits the generalizability of the results, making it challenging to determine which devices are most suitable for continuous cardiac monitoring across diverse patient populations, including the elderly and those with disabilities.

## 5. Discussion

Textile-based ECG sensors and portable ECG devices represent a significant leap forward in non-invasive, wearable cardiac monitoring, offering the potential for continuous, remote tracking of heart health. A recent study suggested that wearable devices for AF screening could have a cost-effectiveness ratio of up to USD 57,824 per quality-adjusted life year (QALY) [42]. It also demonstrated that screening strategies targeting individuals aged 50 years or older can remain cost-effective, though the incremental cost-effectiveness ratio (ICER) increases as the target age decreases. The findings highlight the importance of combining sensitive, scalable initial screening tools with confirmatory testing to maximize resource efficiency. In the case of aforementioned sensors, conductive polymer-based textile electrodes present a promising avenue for cost-effective AF screening technologies due to their low processing costs, durability and flexibility [43]. Polymers like PEDOT-PSS combine electrical conductivity with textile properties through efficient coating methods, enabling scalable production.

The growing aging population and the rise in chronic diseases, especially in remote areas, highlight the need for timely medical care [20]. However, the 2022 US Preventive Services Task Force found insufficient evidence to support widespread AF screening in asymptomatic patients and in populations aged 50 and older. These devices face critical challenges that must be addressed for effective clinical application. Conventional ECG detection methods often struggle with the unconventional limb-lead placements typical of wearable sensors, making robust, real-time algorithms essential for accurate data collection under various conditions [6]. Signal quality is frequently impacted by factors such as body mass, daily movements and anatomical differences between individuals, particularly in electrode–skin contact stability and noise reduction during activities.

In reviewing the included trials, several limitations stand out, highlighting areas for improvement in the future. A significant concern is that only the NOMED-AF TECH trial included patients with disabilities, emphasizing a notable gap in research. This raises important questions about the accessibility and usability of these wearable ECG devices for individuals with mobility or sensory impairments—a demographic that represents roughly 60% of post-stroke patients [17]. The exclusion of this group in most trials limits the understanding of how effectively these devices can support those who may benefit the most from continuous monitoring. Additionally, while the garments used in these trials are often washable, the electrodes are not waterproof, resulting in frequent interruptions to monitoring when the garments need maintenance. This compromises the ability to gather continuous, reliable data, which is particularly crucial for detecting PAF. The aforementioned trial presented a solution to this issue, using two recorders consecutively.

Beyond device-specific limitations, trial design issues such as small sample sizes hinder the generalizability of findings and weaken statistical power, reducing the reliability of conclusions drawn. A larger sample size is necessary to validate the effectiveness of wearable devices across diverse patient populations and their current absence restricts confidence in the applicability of these devices on a broader scale. Additionally, most trials had relatively short monitoring times, with even those extending up to 90 days, potentially being insufficient to capture intermittent AF episodes, a goal Pagola et al. still aim to achieve [29]. Collectively, these limitations point to a clear need for more inclusive, robustly designed studies that can better address usability, practicality and efficacy, particularly for those with accessibility needs and within more extended monitoring frameworks.

On our part, certain limitations must be acknowledged. This review was conducted as a literature review, with more detailed meta-analysis planned for a future systematic review. Its purpose is only to provide foundational context for the topic of ECG textile-based wearables. Additionally, the authors focused on individuals aged 60 and older, even though clinical guidelines recommend AF screening starting at the age of 65 [44]. Some studies suggest a slight lowering of this threshold, finding a significant increase in AF prevalence between the ages of 60 and 65 [43]. As mentioned before, the US Preventive Services Task Force found insufficient evidence to support screening in individuals aged 50 and older, which influenced our decision, as we tried to find the middle ground [20]. Future research should explore the inclusion of younger populations in AF screening. Despite these limitations, this review highlights the potential of wearable ECG technologies in AF screening and sets the stage for future studies. We remain optimistic that ongoing advancements in wearable health technologies will pave the way for broader, more inclusive screening protocols, ultimately improving early detection and patient outcomes.

Addressing usability, anatomical compatibility and patient compliance, as well as enhancing noise filtration and signal accuracy, will be essential in establishing textile ECGs and portable devices as dependable clinical tools for early diagnosis, continuous monitoring and remote cardiac care.

## 6. Conclusions

In conclusion, the development of textile-based electrodes and wearable ECG devices represents a significant advancement in non-invasive, long-term health monitoring, particularly for conditions such as AF. The integration of materials such as conductive polymers and nanofibers into wearable garments offers a promising solution to overcome the limitations of traditional electrodes, providing greater comfort, durability and user compliance. While challenges such as maintaining consistent skin contact impedance and minimizing motion artifacts remain, ongoing innovations in materials and electrode design are steadily improving the performance and feasibility of these systems.

## Figures and Tables

**Table 1:** MERSQI scores of included studies.

Study	Score	Q1 *	Q2 *	Q3 *	Q4 *	Q5 *	Q6 *	Q7 *	Q8 *	Q9 *	Q10 *
[17]	16.00	2	1.5	1.5	3	1	2	1	2	1	1
[29]	15.50	2	1.5	1	3	1	2	1	2	1	1
[30]	15.00	2	0.5	1.5	3	1	2	1	2	1	1
[11]	15.00	2	0.5	1.5	3	1	2	1	2	1	1

Abbreviations: MERSQI—Medical Education Research Study Quality Instrument; * Questions 1–10 of the MERSQI scale. For all included studies, MERSQI scores are listed, including the sums of the ten questions.

**Table 2:** Demographics.

Ref.	[11]	[30]	[29]	[17]
Patients (n)	18	67 *	163	3014
Sex (m)	14	53	102	1535
Age (years)	66 ± 11	63.1 ± 10.6	74 ± 3.5	77.5 ± 7.9
Paroxysmal AF (n)	8	46	64	192
Main inclusion criteria	Admitted for catheter ablation	Post-first catheter ablation for AF	Cryptogenic stroke diagnosis	Geriatric patients

Abbreviations: Ref.—reference; n—number; m—male; AF—atrial fibrillation; * out of 67 patients, 35 were part of the garment group.

**Table 3:** Sensors design and components.

Ref.	[11,41]	[30]	[29]	[17]
Type	Smart bra-type device	Garment ECG device (a stretchy fabric used for sportswear)	Textile wearable Holter elastic vest	Mobile long-term ECG vest
Conductive material	PEDOT-PSS		Elastomeric polymers	N/A
Features	Woven nanofibers coated with PEDOT-PSS; an insulated electroconductive lead ribbon connected between the electrodes and a connector terminal for signal transmission	Lead wires pre-installed on the wearable device;	The ECG signal is stored in a digital memory card inserted into a signal recorder	Exchangeable recorders fixed to the vest, a docking station acting as a charger, a GSM transmitter and a patient monitoring platform for data analysis and storage
No. of ECG Leads	1	1	2	2
Wireless communication protocol	Bluetooth		N/A	3G, WiFi, LAN
Total analysis time (hours)	3.05–3.85	218.4–292.8	1642	357.6–693.6
Energy supply autonomy (hours)	N/A	336	22	>24
Patient modules	Simultaneous positioning of textile and gel electrodes	Polyester nanofiber fabric coated with a dielectric polymer	High-quality and comfortable elastic fabric without adhesives	Two recorders to maintain continuous ECG acquisition (during charging)
Manufacturer	Toray Industries Inc. (Tokyo, Japan) and Cardyrode-P^®^, SUZUKEN Co., Ltd. (Nagoya, Japan)	Toray Industries, Inc. (Tokyo, Japan)	Nuubo^®^ (Madrid, Spain)	Comarch Healthcare (Krakow, Poland)

Abbreviations: Ref.—references; ECG—electrocardiogram; N/A—not available; PEDOT-PSS—poly(3,4-ethylenedioxythiophene) poly(styrenesulfonate); GSM—Global System for Mobile communication.

**Table 4:** Technical performance indicators.

Ref.	[11]	[30]	[29]	[17]
Total analysis time (hours)	3.05–3.85	218.4–292.8	1642	357.6–693.6
SNR	Lower	Lower	N/A	Higher
Signal stability	N/A	82.4	N/A	95.8
Sensitivity to detect AF (%)	N/A	N/A	89.6	93
Specificity to detect AF (%)	N/A	N/A	27.59	85

Abbreviations: Ref.—references; SNR—signal-to-noise ratio; AF—atrial fibrillation; N/A—not available.

**Table 5:** Usability.

Ref.	[11]	[30]	[29]	[17]
User state	Resting and walking			Resting, walking, exercising
Discomfort during resting/physical activities	Mild			
Patients with no adverse effects	94.44%	97.6%	N/A	79.3%
Redness	0.0%	2.3%	N/A	9.4%
Rash	0.0%	0.0%	N/A	3.7%
Skin chafing	0.0%	0.0%	N/A	3.3%

Abbreviations: Ref.—references; N/A—not available.

## Data Availability

Data available upon request.

## Abbreviations

AF—Atrial fibrillation; AgNW/TPU—silver nanowire/thermoplastic polyurethane; CVDs—cardiovascular diseases; ECG—Electrocardiography; FDA—Food and Drug Administration; HR—heart rate; ICER—the incremental cost-effectiveness ratio; ICM—implantable cardiac monitors; ICMs—implantable cardiac monitors; MERSQI—Medical Education Research Study Quality Instrument; MeSH—Medical Subject Headings; MWCNTs—multiwalled carbon tubes; N/A—not available; PAF—paroxysmal AF; PDMS—polydimethylsiloxane; PVDF—polyvinylidene fluoride; RCT—randomized clinical trial; Ref.—references; SECP—skin–electrode contact pressure; SNR—signal-to-noise ratio; WHO—World Health Organization.
